# Nonstandardized High-Intensity Dialysis Dose Improves Survival in Patients With End-Stage Renal Disease

**DOI:** 10.7759/cureus.71725

**Published:** 2024-10-17

**Authors:** Diana D Nenova, Yanko G Yankov, Gergana M Chausheva

**Affiliations:** 1 Clinic of Nephrology and Dialysis, University Hospital St. Marina, Varna, BGR; 2 Department of Internal Disease, Medical University "Prof. Dr. Paraskev Stoyanov", Varna, BGR; 3 Clinic of Maxillofacial Surgery, University Hospital St. Marina, Varna, BGR; 4 Department of General and Operative Surgery, Medical University "Prof. Dr. Paraskev Stoyanov", Varna, BGR; 5 Central Clinical Laboratory, University Hospital St. Marina, Varna, BGR; 6 Department of Clinical Laboratory, Medical University "Prof. Dr. Paraskev Stoyanov", Varna, BGR

**Keywords:** adequacy, death, dialysis, improvement, malnutrition, mortality, non-standardized, quality of life, risk, survival

## Abstract

Introduction

One of the most important critical determinants of quality of life and adequacy of hemodialysis (HD) performed in patients is the recorded survival and mortality rates. Nowadays, as an adequately performed HD dialysis, we accept the one with reaching values for the index single pool Kt/V (spKt/V) higher than 1.2. In recent years, more intensive HD regimens with spKt/V≥1.5 have been increasingly discussed, which can significantly improve patient survival. However, their benefit has yet to be proven, as extremely high spKt/V values can be misleading as they may result from malnutrition leading to reduced survival and increased relative risk of death. The aim of this study is to present to the community the impact of nonstandardized high dialysis doses (spKt/V>1.5) on annual survival and mortality rates in the dialysis population and to explore new strategies for enhancing quality of life and survival, as well as to promote their regular implementation in clinical practice and personalized patient care.

Material and methods

The present retrospective study was conducted at the Clinic of Nephrology and Dialysis at University Hospital St. Marina in Varna, Bulgaria for a period of five years. It involved a survival analysis of 100 dialysis patients who met the inclusion and exclusion criteria. The dialysis dose delivered was the criterion for their allocation into three studied groups: Group 1 with adequate dose (standardized) - spKt/V = 1.2-1.49, Group 2 with high dose (nonstandardized) - spKt/V≥1.5, and Group 3 with inadequate dose (low) - spKt/V≤1.19. We recorded total annual mortality and survival rates, analyzing their relationship with the delivered dialysis dose and assessing the relative risk of death and expected survival.

Results

The analysis results indicated that high-intensity regimens with an spKt/V≥1.5 were linked to a better patient prognosis, with a significantly lower risk of death compared to standard regimens and increased survival expectancy. Data from the survival analysis suggested that the long-term impact of increasing the dialysis dose (spKt/V≥1.5) on survival becomes evident after the third year. Additionally, nutritional status parameters emerged as key risk factors for deterioration, along with the indicators of dialysis adequacy.

Discussion

Improved survival rates have been observed in patients undergoing nocturnal HD. In the latter, significantly higher spKt/V values have been achieved due to extended dialysis sessions, as well as in those performing dialysis at home. Despite concerns about possible misinterpretation of extremely high spKt/V values (>1.5) as a sign of malnutrition when urea volume of distribution is reduced, it is found that when only urea clearance (Kt) is used, without volume counts, the risk of death decreases by 2% for each liter increase in clearance. This demonstrates that the assessment of dialysis adequacy is a much broader concept than the values of generally accepted indicators and should be focused on individualized care and risk assessment tailored to each patient.

Conclusions

We believe that the modern nephrological community should strive to achieve a high dialysis dose (spKt/V≥1.5) to improve clinical outcomes and patient prognosis. Assessing dialysis adequacy is complex and goes beyond a simple numerical value such as spKt/V. It requires careful monitoring of nutritional status and the management of all HD-related complications.

## Introduction

Critical determinants of quality of life and dialysis adequacy in the increasing population of hemodialysis (HD) patients are recorded survival and mortality rates. These rates should be interpreted alongside validated clinical outcome indicators. Currently, single pool Kt/V (spKt/V)>1.2 is established as the threshold for adequate dialysis per Kidney Disease Outcomes Quality Initiative (KDOQI) 2015 recommendations [1]. In recent years, more intensified HD regimens with spKt/V≥1.5 have also been discussed, which can significantly improve survival [2].

However, some authors argue that extremely high spKt/V values may be misleading, attributing them to malnutrition, which reduces the urea volume of distribution (V) in the spKt/V calculation. This malnutrition can result in decreased survival and an increased relative risk of death, despite the apparent high delivered dialysis dose [2-7].

It is alarming that in recent years increasingly high mortality rates have been reported despite the improvement of dialysis technologies and the improved biocompatibility of dialysis membranes. This, together with the ever-increasing incidence of chronic kidney disease (CKD) and those in need of renal replacement therapy, necessitates urgent measures to improve the survival and quality of life of these patients.

The aim of this study, which is based on research information presented in a doctoral dissertation, is to determine the impact of nonstandardized high dialysis doses (spKt/V>1.5) on annual survival and mortality rates in the dialysis population. The goal is to explore new strategies for enhancing quality of life and survival, as well as to promote their regular implementation in clinical practice and personalized patient care.

## Materials and methods

This is a retrospective study, which was conducted at the Clinic of Nephrology and Dialysis at University Hospital St. Marina in Varna, Bulgaria for a period of five years (from January 2017 to December 2021). It involved a survival analysis of all 100 dialysis patients who met the including and excluding criteria we set. The dialysis dose delivered was the criterion for their allocation into three studied groups: Group 1 with adequate dose (standardized per KDOQI 2015 criteria) - spKt/V = 1.2-1.49, Group 2 with high dose (nonstandardized) - spKt/V≥1.5, and Group 3 with inadequate dose (low) - spKt/V≤1.19. In the last group, only patients with normal nutritional status (normalized protein catabolic rate (nPCR) >1.2) were included to avoid the effect of malnutrition on spKt/V and errors in the interpretation of the result. For the specified five-year period, total annual mortality and survival were recorded, and their relationship to dialysis dose delivered in the total sample and the formed groups was assessed, with an analysis of the relative risk of death and expected survival.

Inclusion criteria mandated that participants be at least 18 years old, with no upper age limit, and have undergone chronic HD for over six months, with minimal residual renal function defined as diuresis below 100 ml per day, and their medical documentation was complete. Exclusion criteria ruled out individuals under 18, with no upper age limit, those with malignancies, patients with poor nutritional status (nPCR<1.2) in the high-dose group, and patients with incomplete medical documentation.

As a part of the study, the medical records of 128 patients were reviewed, of which 112 met the study criteria. Out of these, 100 patients who fulfilled the inclusion and exclusion criteria were analyzed and statistically processed. Twelve patients, with an average age of 56.4 ± 4.9 years (38.4% women and 61.6% men), were excluded from the cohort. Among them, eight had a malignant condition, and four had poor nutritional status with an nPCR below 1.2 in the group with spKt/V≥1.5.

Fresenius Medical Care series 4008 and 5008 machines (Fresenius Medical Care AG & Co. KGaA, Bad Homburg, Germany) were utilized across various dialysis dose regimes, averaging 11.38 ± 1.3 hours per week for Group 3, 15.21 ± 0.4 hours for Group 2, and 16.1 ± 0.2 hours for Group 1. Treatments employed bicarbonate dialysate with a dialysate flow rate (Qd) of 500-800 ml/min and a blood flow rate (Qb) of 200-400 ml/min, tailored to the vascular access available. Polysulfone dialyzers were used: type “Etropal” (Etropal JSC, Etropole, Bulgaria), “Diadema” (Etropal JSC), “Ashahi” (Asahi Kasei Medical Co., Ltd., Tokyo, Japan), “Fresenius F7” (Fresenius Medical Care AG & Co. KGaA) with a surface area of 1.6-2.1 m^2^.

The study evaluated dialysis adequacy indicators - single pool Kt/V (spKt/V) and urea reduction ratio (URR%) - alongside nutritional status measures, including nPCR and serum albumin. The dose regimen was intensified during the study by increasing the average weekly dialysis time in the three-dose regimens mentioned above, increasing the blood flow rate to 400 ml/min and dialysate flow to 800 ml/min, and enhancing the permeability or surface area of dialyzers.

According to the National Health Insurance Fund, requirements of Bulgaria biochemical laboratory tests, including measurements of urea, creatinine, and albumin, were conducted routinely for all studied patients. Standardized conditions were used to collect blood samples to ensure the accuracy of the results. A coupled enzyme reaction with glutamate dehydrogenase through a UV kinetic method on the ADVIA Chemistry 1800 system (Siemens, Germany) was used to determine urea levels (mmol/l) with reference ranges (RRs) set at 3.2-8.2 mmol/l. The Jaffe-kinetic method on the same system was used to determine creatinine levels (mmol/l), with RRs of 44-115 mmol/l. The assessment of albumin levels (g/l) used a colorimetric method with bromocresol green on the same system, with RRs of 32-48 g/l.

To minimize recirculation effects and urea rebound, the stop-pump technique was employed before and/or after HD. Serum albumin samples were routinely taken prior to the initiation of the dialysis process to avoid ultrafiltration (UF) impact. For assessment of dialysis adequacy and nutritional status indicators, urea and creatinine samples were collected both pre- and post-dialysis. Table 1 presents the formulas used for the calculation of spKt/V, URR%, and nPCR.

**Table 1:** Mathematical methods Age: age in years; C: post-dialysis urea nitrogen; Cn: pre-dialysis urea nitrogen from the next hemodialysis; Co: pre-dialysis urea nitrogen; H: height in cm; ID: inter-dialysis time in hours (h); ln: natural logarithm; nPCR: normalized protein catabolic rate; R: C/Co; spKt/V: single-pool Kt/V; t: duration of dialysis in hours (h); TBW: volume of total body water; UF: applied ultrafiltration in liters (l); URR%: urea reduction ratio; W: patient’s optimal weight after dialysis in kg

Indicator	Formula
spKt/V	spKt/V = -ln(R-0.008xt) + [4-3.5 x R] x 0.55 UF/W)
URR%	URR% = 100 × (1–C/Co)
nPCR	nPCR = 0.22 + 0.36 × (Cn-C) × 24/ID
TBW men	TBW = 2.447-0.09516 × Age + 0.1074 × H + 0.3362 × W
TBW women	TBW = -2.097 + 0.1069 × H + 0.2466 × W

The statistical analysis was conducted using IBM SPSS Statistics for Windows, Version 20.0 (Released 2011; IBM Corp., Armonk, NY, USA) on a Windows 10 platform (Microsoft Corporation, Redmond, Washington, USA). The analysis methods included descriptive statistics to determine the mean levels and variability of quantitative variables, along with absolute and relative values for qualitative variables. Parametric tests (ANOVA) and non-parametric tests (Chi-square) were used for hypothesis testing. Correlation analysis (Pearson’s r) assessed the relationships between observed phenomena, while receiver operating characteristic (ROC) curve analysis evaluated the predictive value of the studied variables. Area under the curve (AUC) and 95% CIs were used in the analysis. Expected survival rates were estimated using Kaplan-Meier curves, and Cox regression was applied to estimate the proportional hazard ratio (HR) of death based on dialysis dosage. Results with p-values lower than 0.05 were deemed statistically significant.

## Results

The mean age of the study cohort at the start was 58.55 ± 11.84 years, with 40% women and 60% men. The mean age of the study cohort at the end of the study was 56.9 ± 11.23 years, with 38.2% women and 61.8% men.

Analyses of variation and ANOVA (Table 2) were conducted for dialysis adequacy indicators (spKt/V and URR%) as well as nutritional status measures (nPCR and serum albumin) during the review period.

**Table 2:** Results of ANOVA and analysis of variance over a five-year period Alb (g/l): serum albumin; nPCR (g/kg/d): normalized protein catabolic rate; spKt/V: single-pool Kt/V, dialysis adequacy index; URR%: urea reduction ratio

Indicator (X ± SD)	2017 (n = 100)	2018 (n = 91)	2019 (n = 84)	2020 (n = 80)	2021 (n = 66)	ANOVA (F)	p-value
spKt/V	1.25 ± 0.2	1.34 ± 0.15	1.39 ± 0.2	1.54 ± 0.31	1.59 ± 0.32	27.4298	<0.0001
URR %	65.59 ± 7.23	71.12 ± 5.7	71.06 ± 6.32	73.02 ± 7.62	73.57 ± 7.26	19.2577	<0.0001
nPCR (g/kg/d)	1.09 ± 0.14	1.16 ± 0.12	1.18 ± 0.12	1.21 ± 0.15	1.22 ± 0.14	12.57039	<0.0001
Alb (g/l)	34.38 ± 4.71	36.64 ± 4.18	37.46 ± 4.53	37.73 ± 5.14	37.02 ± 4.34	7.90568	<0.0001

The trend toward increasing the dialysis dose, reflected in the spKt/V and URR values, was notable. At the beginning of the study, the mean spKt/V was 1.25 ± 0.2 and the URR was 65.59 ± 7.23%. By the end of the five-year follow-up, these values had risen to 1.59 ± 0.32 and 73.57 ± 7.26%, respectively. Additionally, there was a positive trend in nutritional status, with the mean nPCR increasing from 1.09 ± 0.14 at the study’s start to 1.22 ± 0.14 by the end. Regarding serum albumin, after the significant increase in the first year of the study from 34.38 ± 4.71 to 36.64 ± 4.18, no significant change was observed in subsequent years.

**Figure 1:**
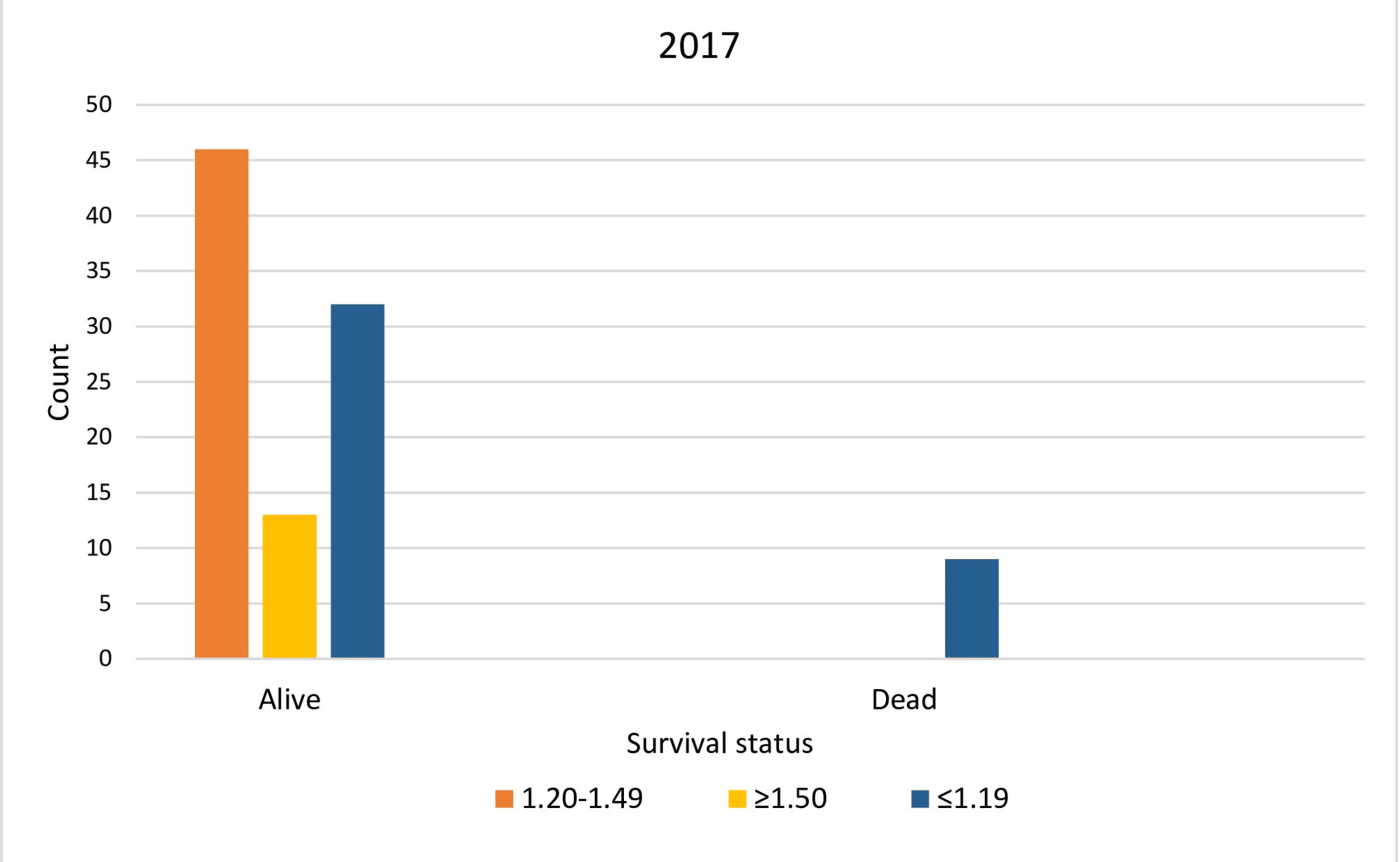
Achieved survival levels of the studied population for 2017 Orange column: Group 1 (spKt/V = 1.2-1.49); yellow column: Group 2 (spKt/V≥1.5); blue column: Group 3 (spKt/V≤1.19)

In 2017, with an average annual delivered spKt/V of 1.25 ± 0.2, the survival rate in Group 1 (standard dialysis dose) was the highest at 50.5%. Although Group 2 (high dialysis dose) had a survival rate of only 14.3%, this appeared significantly lower primarily due to the smaller sample size. In practice, in both Group 1 and Group 2, the recorded survival rates were 100%. The strong association between inadequate dialysis dose and mortality was proved by Chi-square tests (χ²) (χ² = 14.2, p < 0.001) (Table 3, Figure 1).

**Table 3:** Association between mortality and achieved dialysis dose for 2017 (Chi-squared test), χ² = 14.2, p < 0.001

Survival status		Group 1	Group 2	Group 3	Number of studied patients
Alive	Count	46	13	32	91
	Percentages (%)	50.5 %	14.3 %	35.2 %	100%
Dead	Count	0	0	9	9
	Percentages (%)	0%	0%	100%	100%
Total	Count	46	13	41	100
	Percentages (%)	46%	13%	41%	100%

Cox regression analysis revealed that the risk of death in Group 3 was 2.11 times higher than in Group 1 (standard dialysis dose) with an HR of 2.11 (95% CI = 1.32-3.39, p = 0.002). In contrast, Group 2 (high dialysis dose) showed no statistically significant difference in mortality risk compared to Group 1, with an HR of 1.03 (95% CI = 0.55-1.93, p = 0.917).

**Figure 2:**
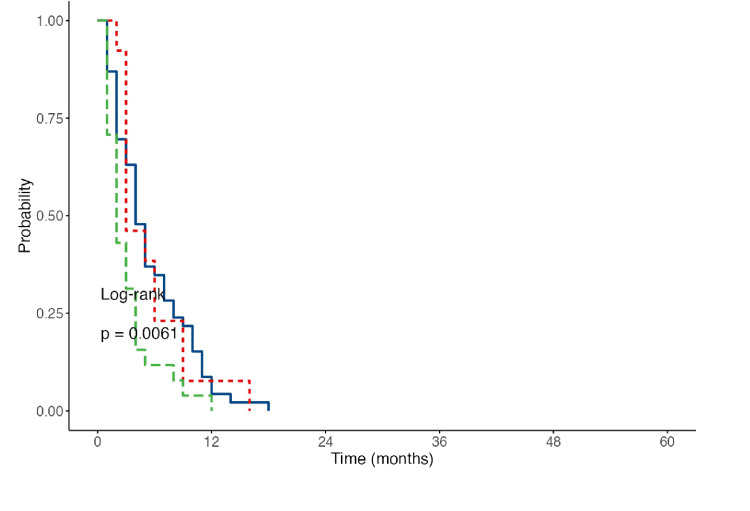
Curves of the expected survival of the studied patients at different dose regimens of HD for 2017 Blue line: expected survival for Group 1; red line: expected survival for Group 2; green line: expected survival for Group 3 HD: hemodialysis

Kaplan-Meier survival curves were created, illustrating that in the 2017 sample, Group 1 (spKt/V = 1.2-1.49) had the highest expected survival at 18 months. Group 2 followed closely, with an expected survival of 16 months (Figure 2). Notably, the curve for Group 2 was relatively steeper, stabilizing around the sixth month of treatment, whereas Group 1 exhibited a significantly steeper decline during the first 12 months. This indicates that Group 1 experienced a longer critical period for adverse events, with stabilization occurring after the 12th month.

The analysis results for 2018 (χ² = 31.6, p < 0.001) and 2019 (χ² = 31.1, p < 0.001) showed no significant differences from the initial year of the study, even as the average annual dialysis dose increased to spKt/V = 1.34 ± 0.15 and spKt/V = 1.39 ± 0.2. Patients in Group 1 continued to exhibit the best survival prognosis, life expectancy, and risk of death.

However, survival analysis results changed notably in the last two years, with average annual dialysis doses increasing to spKt/V = 1.54 ± 0.31 in 2020 and spKt/V = 1.59 ± 0.32 in 2021. The annual mortality rate in the cohort for the same period significantly rose due to COVID-19 infections and complications, which, as a respiratory illness, did not correlate with the dialysis dose (p > 0.05). To ensure objectivity in the survival analysis, relevant COVID-19-related events were excluded from the sample.

**Figure 3:**
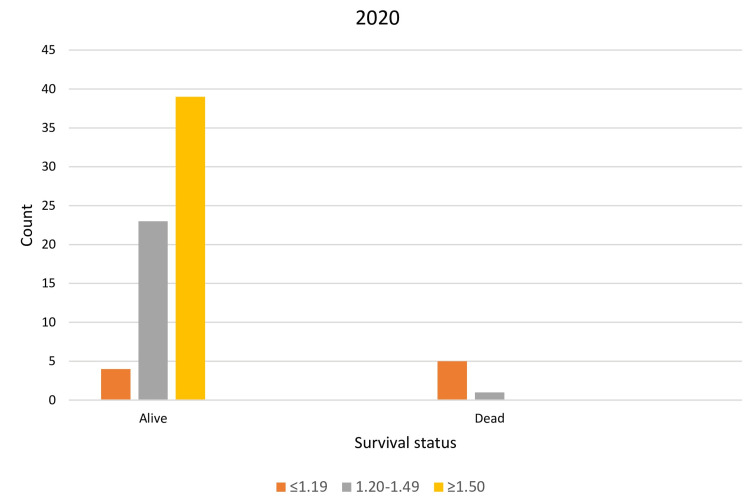
Achieved survival levels of the studied population for 2020 Gray column: Group 1 (spKt/V = 1.2-1.49); yellow column: Group 2 (spKt/V≥1.5); orange column: Group 3 (spKt/V≤1.19)

**Figure 4:**
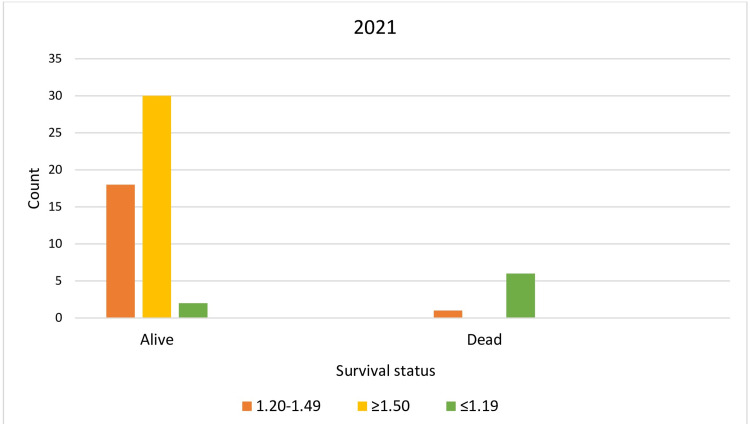
Achieved survival levels of the studied population for 2021 Orange column: Group 1 (spKt/V = 1.2-1.49); yellow column: Group 2 (spKt/V≥1.5); green column: Group 3 (spKt/V≤1.19)

Chi-square analysis again confirmed a strong association between mortality and Group 3 (low dialysis dose). However, with increasing dialysis doses, survival rates shifted in favor of Group 2 (high dialysis dose) (p < 0.001) (Table 4, Table 5, Figure 3, Figure 4). This trend was attributed to longer dialysis durations and improved patient rehabilitation, highlighted by a weak positive correlation (r = 0.41, p = 0.01) between the last and delivered dialysis doses.

**Table 4:** Association between mortality and achieved dialysis dose for 2020 (Chi-squared test)

Survival status		Group 1	Group 2	Group 3	Number of studied patients
Alive	Count	39	23	4	66
	Percentages (%)	59.10%	34.80%	6.10%	100%
Dead	Count	0	1	5	6
	Percentages (%)	0%	16.70%	83.30%	100%
Total	Count	39	24	9	72
	Percentages (%)	54.20%	33.30%	12.50%	100%

**Table 5:** Association between mortality and achieved dialysis dose for 2021 (Chi-squared test)

Survival status		Group 1	Group 2	Group 3	Number of studied patients
Alive	Count	1	0	6	7
	Percentages (%)	14.30%	0%	85.7%	100%
Dead	Count	18	30	2	50
	Percentages (%)	36%	60%	4%	100%
Total	Count	19	30	8	57
	Percentages (%)	33.30%	52.60%	14%	100%

It was assessed, based on Cox regression analysis for 2020, that patients in Group 2, who received a high dialysis dose, experienced better survival rates with a significantly reduced risk of death - HR 0.6 (95% CI = 0.35-1.02, p = 0.051) compared to Group 1. A similar trend was observed in the 2021 sample, where Group 2 had a reduced risk of HR 0.59 (95% CI = 0.32-1.08, p = 0.054).

**Figure 5:**
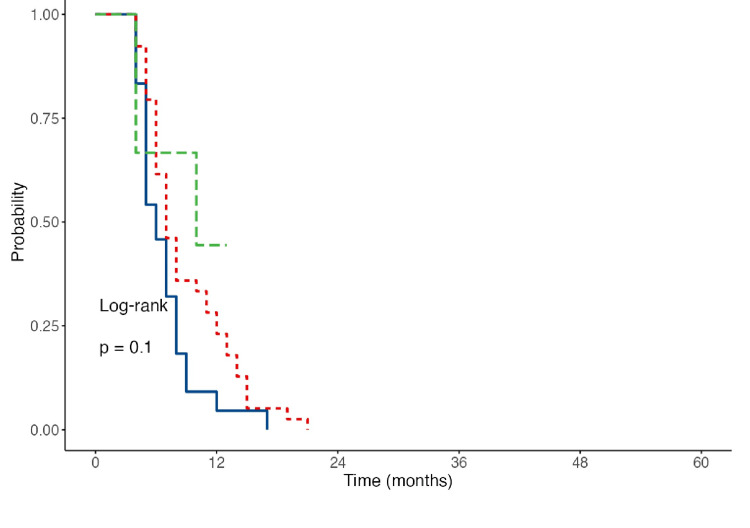
Curves of the expected survival at different dose regimens of HD of the observed population for 2020 Blue line: expected survival for Group 1; red line: expected survival for Group 2; green line: expected survival for Group 3 HD: hemodialysis

**Figure 6:**
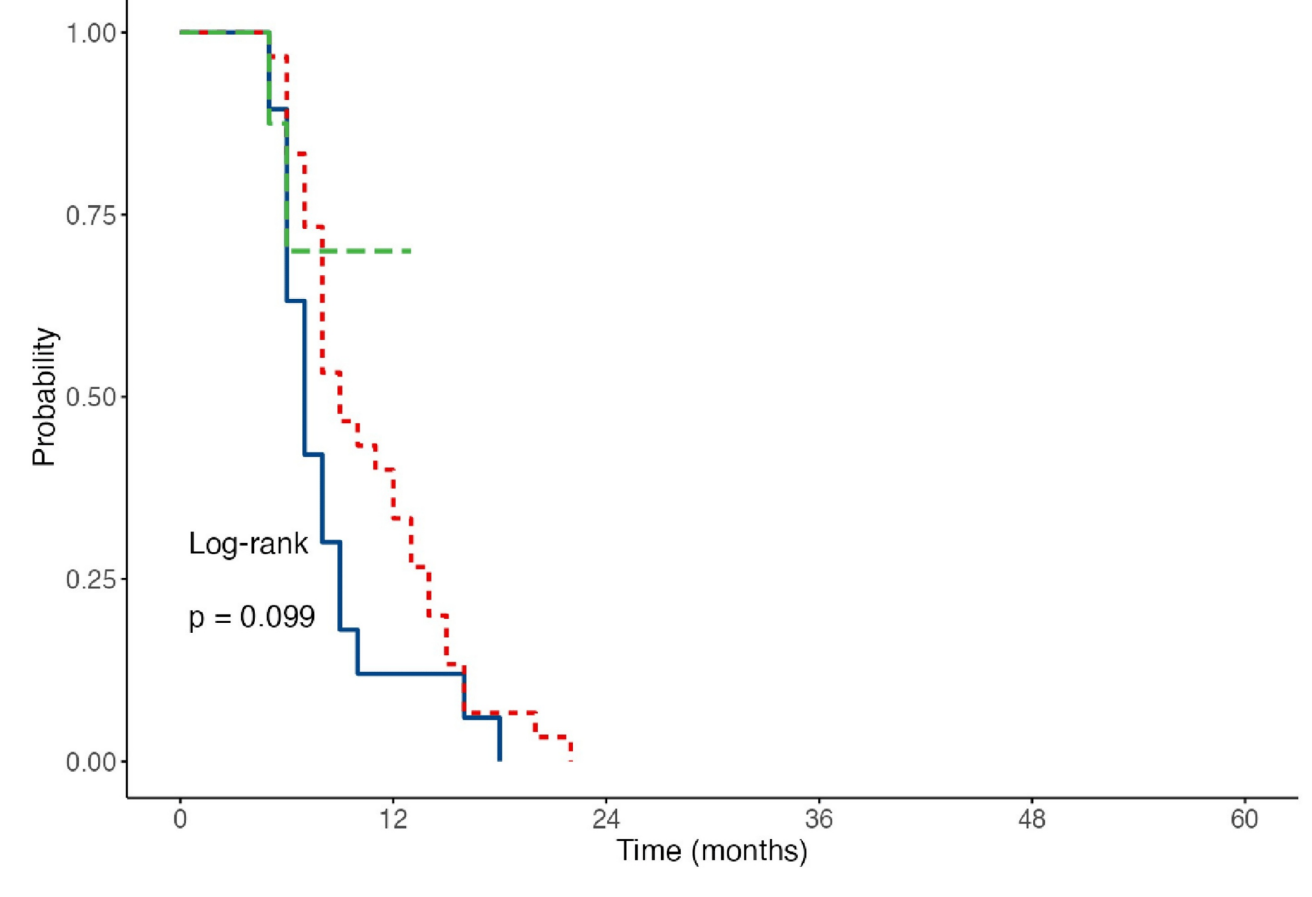
Curves of the expected survival at different dose regimens of HD of the observed population for 2021 Blue line: expected survival for Group 1; red line: expected survival for Group 2; green line: expected survival for Group 3 HD: hemodialysis

Survival curves for the respective periods clearly demonstrated significantly higher expected survival rates for Group 2. As shown in Figure 5 and Figure 6, the expected survival for Group 2 increased to nearly 24 months, contrasting with the 12-16 months expected during the first three years of the study. Thus, it can be concluded that the long-term impact of increased dialysis doses on survival became evident after the third year, leading to a markedly improved prognosis for patients.

**Figure 7:**
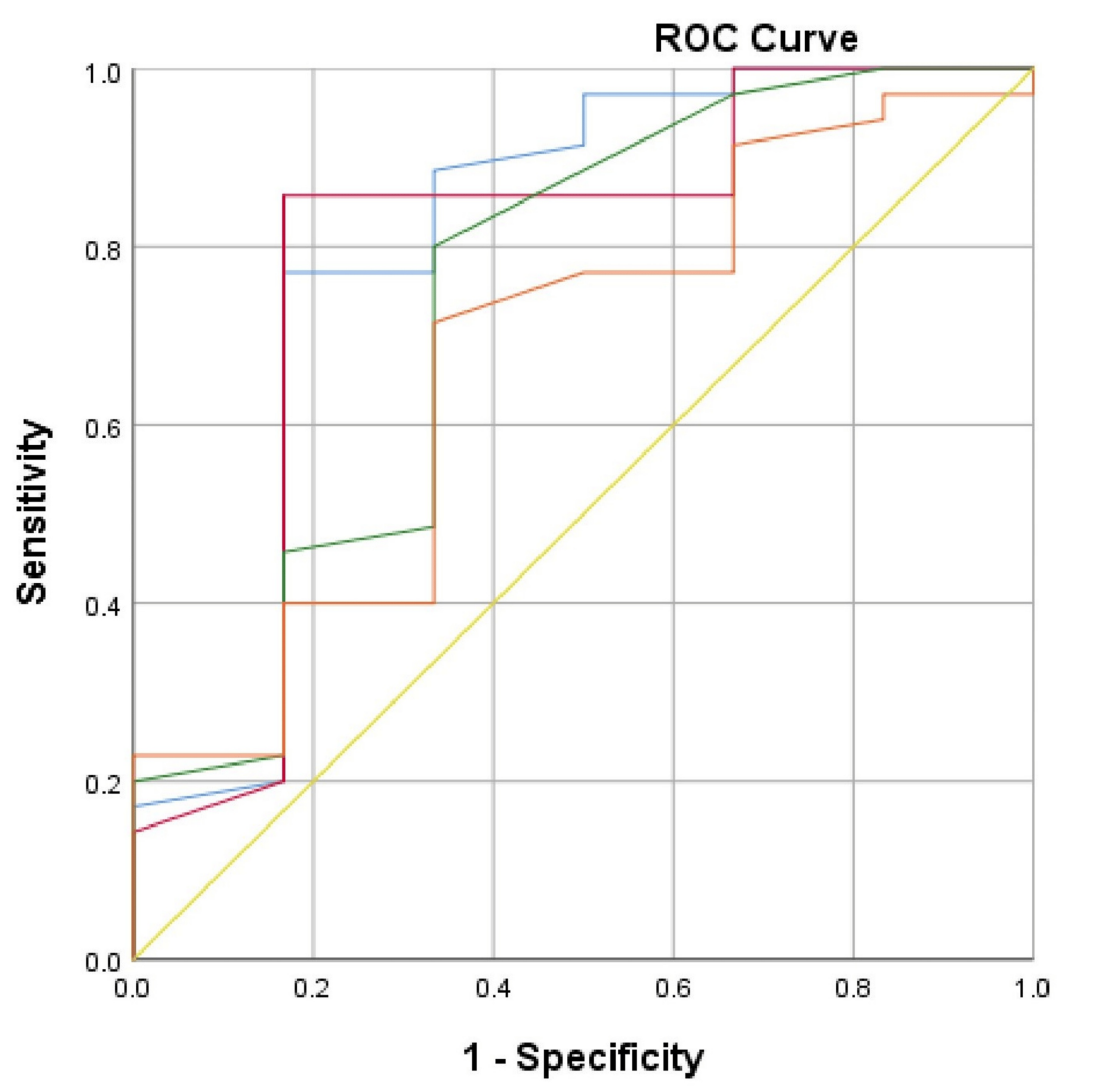
ROC curve for evaluation of the predictors of deteriorating clinical outcome in the studied patients in Group 1 Blue line: spKt/V; red line: URR%; green line: nPCR; orange line: serum albumin; yellow line: reference line ROC: receiver operating characteristic

**Figure 8:**
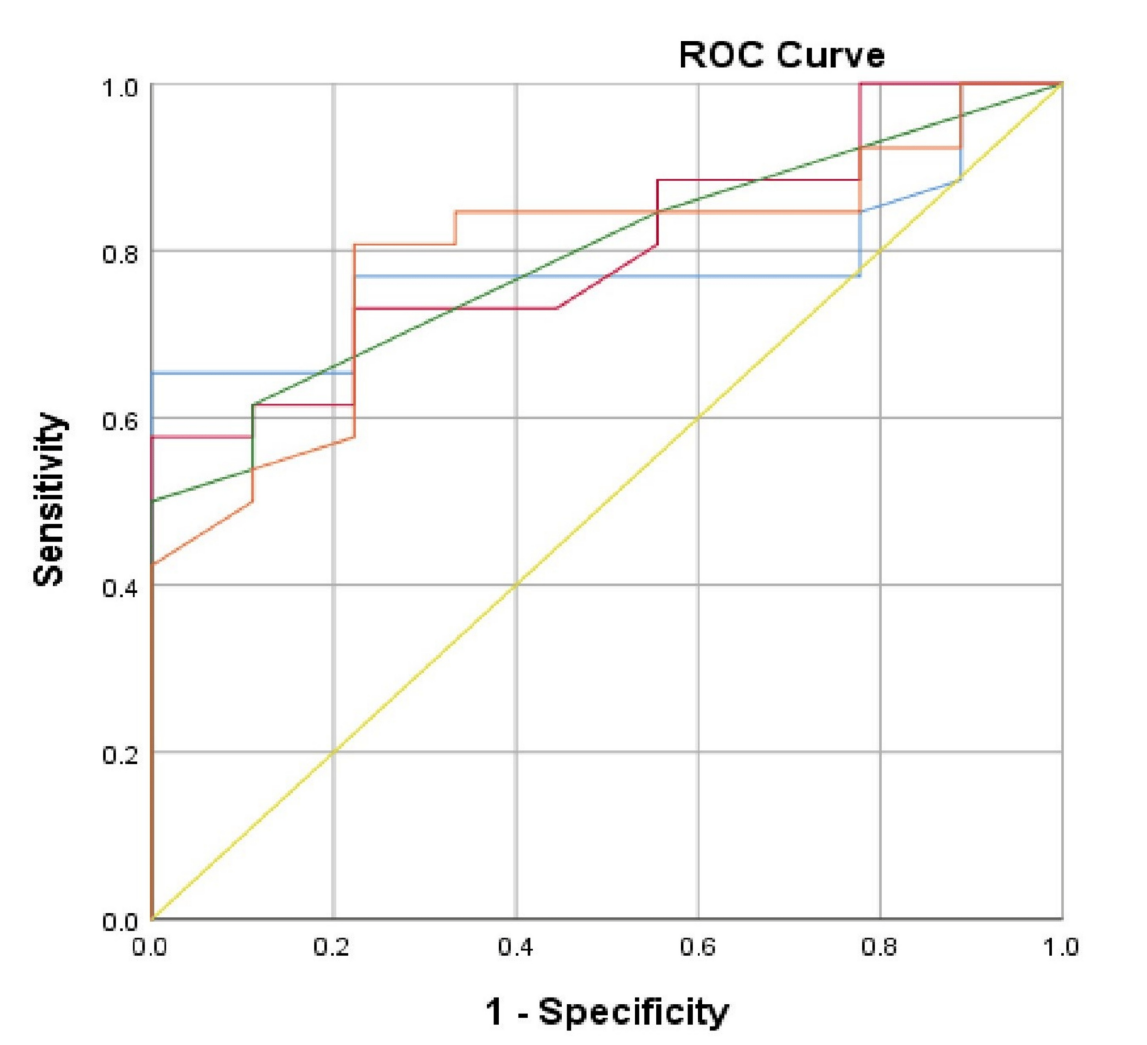
ROC curve for evaluation of the predictors of deteriorating clinical outcome in the studied patients in Group 2 Blue line: spKt/V; red line: URR%; green line: nPCR; orange line: serum albumin; yellow line: reference line ROC: receiver operating characteristic

ROC curves (Figure 7, Figure 8) for Group 1 and Group 2, representing standard and high dialysis doses, highlighted the main predictors of mortality within the sample. A ROC curve was not generated for Group 3, as spKt/V<1.19 and nPCR<1.2 have long been established as indicators of poor prognosis in the HD population.

In Group 1, the primary predictors of death were spKt/V (AUC = 0.81, 95% CI = 0.581-1.00, p = 0.01) and URR % (AUC = 0.782, 95% CI = 0.541-1.00, p = 0.021). Critical values identified were spKt/V<1.21, with a sensitivity of 85.7% and specificity of 33.23%, and URR<70.8%, with a sensitivity of 82.9% and specificity of 16.7%.

For Group 2, alongside spKt/V (AUC = 0.798, 95% CI = 0.668-0.952, p = 0.006) and URR % (AUC = 0.779, 95% CI = 0.632-0.916, p = 0.011), nutritional status indicators were also significant. These included nPCR (AUC = 0.791, 95% CI = 0.654-0.936, p = 0.01) and serum albumin (AUC = 0.794, 95% CI = 0.657-0.938, p = 0.012). The critical values for the studied indicators were spKt/V<1.41 (sensitivity 76.9%; specificity 33.3%), URR<75.1% (sensitivity 65.4%; specificity 22.2%), nPCR<1.2 (sensitivity 62.4%; specificity 11.6%), and serum albumin lower than 35.2 g/l (sensitivity 80.8%; specificity 33.3%).

**Figure 9:**
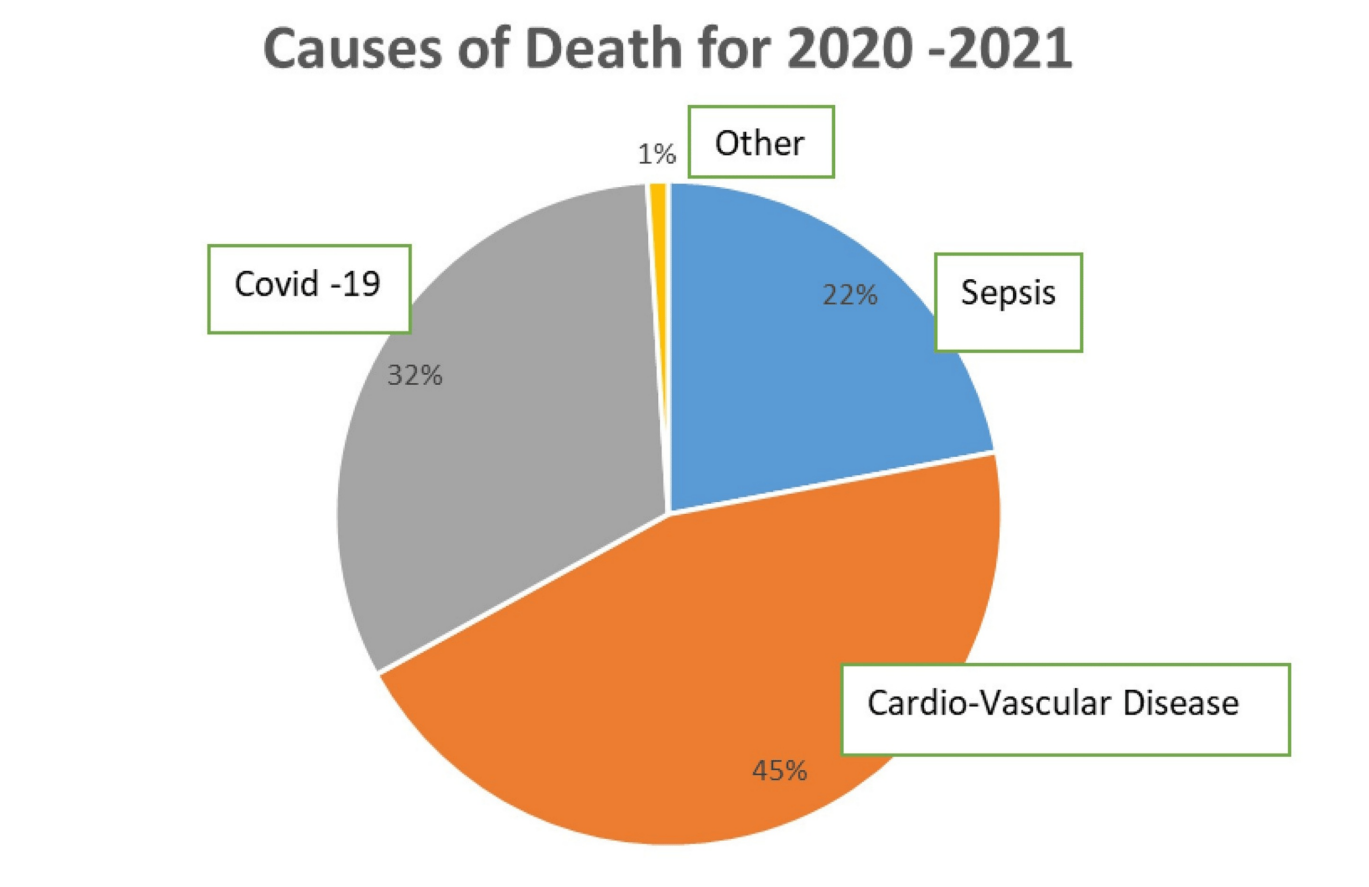
Primary causes of mortality for a five-year follow-up period

Figure 9 illustrates the primary causes of mortality within the studied population.

**Figure 10:**
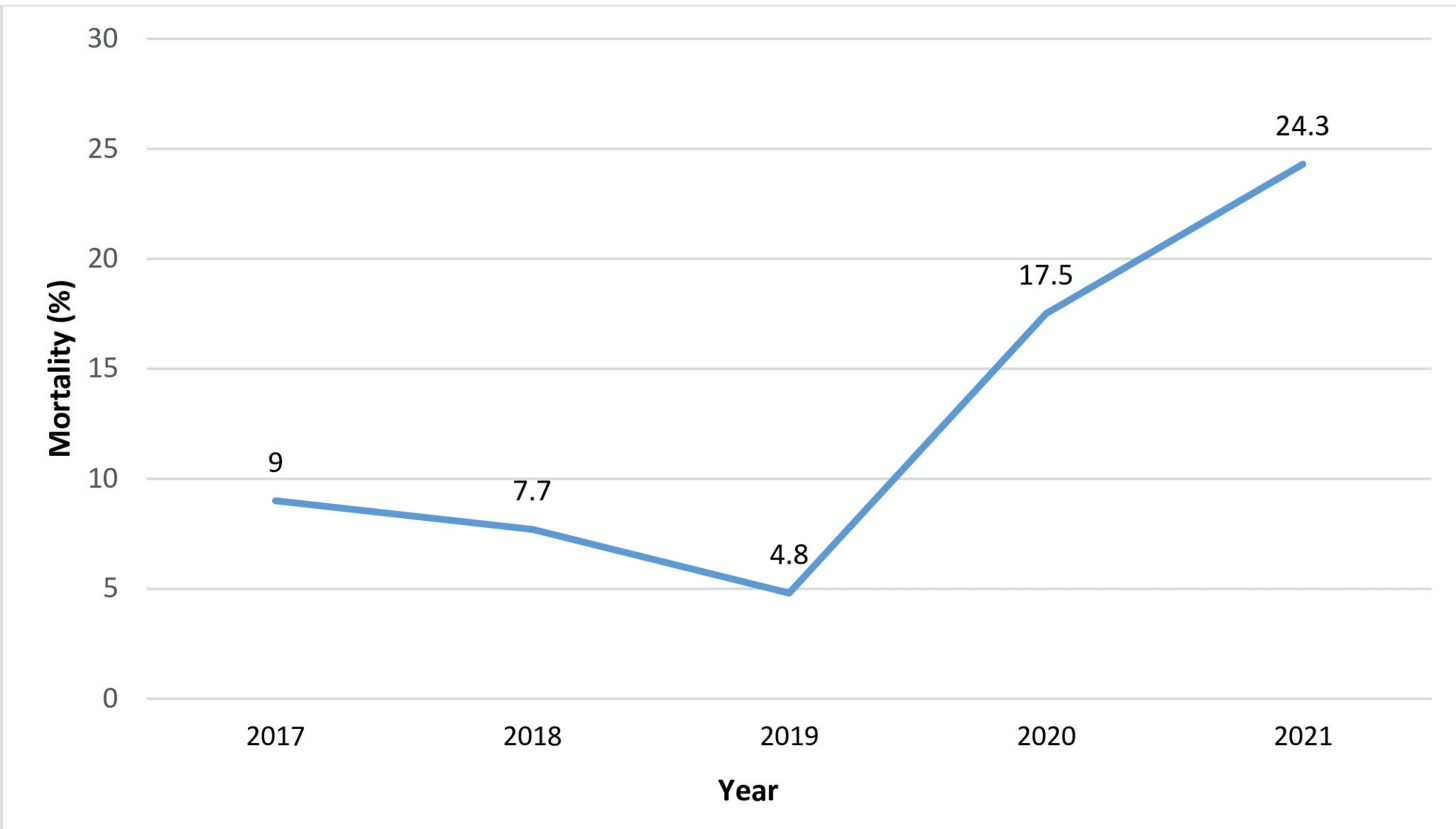
Registered total mortality for the period 2017-2021

As previously noted, the final two years of the study experienced a notable increase in annual mortality rates (Figure 10), primarily due to COVID-19 infections. The data indicated that the pandemic had a severe impact on the dialysis population, contributing to over 50% of the total annual mortality. Specifically, in 2020, COVID-19 accounted for 67.2% of all deaths in the sample, while in 2021, this figure was 66.3%.

**Figure 11:**
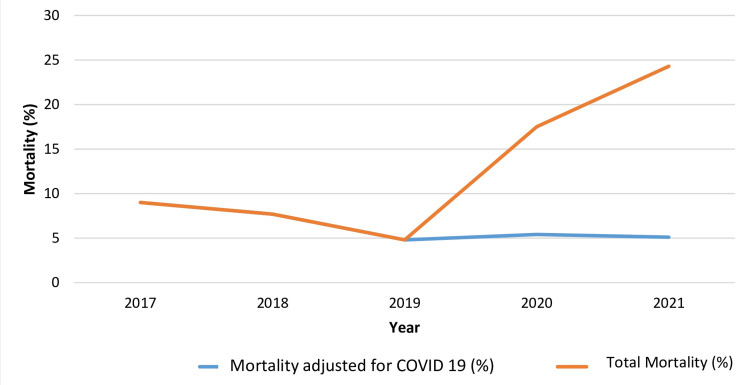
Corrected mortality curve for the period 2017-2021 Blue line: mortality adjusted for COVID-19; orange line: overall mortality in the studied population

The overall mortality in the studied population, adjusted for the impact of COVID-19, is presented in Figure 11.

## Discussion

In recent years, improved life expectancy has been reported in patients with end-stage renal disease (ESRD). According to the 2018 United States Renal Data System report, the adjusted five-year survival rate for patients who began dialysis in 2011 was 42% [8-10]. Currently, an spKt/V>1.2 is established as the threshold for adequate dialysis, with recent discussions emphasizing more intensive HD regimens, which can significantly improve survival rates [1,2]. Similar improvements have been observed in patients undergoing nocturnal HD, where higher spKt/V values are achieved due to longer dialysis sessions, as well as in those utilizing home dialysis [2,11-18]. Besides extending treatment duration, other factors influencing outcomes have gained attention, such as dialysis frequency, volume control, UF rates, and enhanced clearance of medium-sized molecules [1,2].

One ongoing debate in the nephrology community pertains to the impact of high dialysis doses, specifically spKt/V≥1.5, on patient survival and quality of life. Some researchers argue that extremely high Kt/V values (>1.5) or a URR of 75-79% are linked to decreased survival and an increased risk of death [2-5]. They suggest that elevated spKt/V levels may be “illusory,” stemming from malnutrition and increased protein catabolism, which reduces the volume (V) in the spKt/V formula [6,7]. However, studies indicate that using urea clearance Kt while disregarding volume as an alternative measure of dialysis adequacy does not raise the risk of death; rather, it decreases by 2% for every liter increase in clearance [2,7,19,20]. This highlights the need for a multifaceted approach to assess dialysis adequacy, taking into account the corrected risk for individual patients.

Our study findings demonstrate a strong correlation between survival and high dialysis doses of spKt/V≥1.5. The survival analysis showed that as the annual dialysis dose increased from an spKt/V of 1.25 ± 0.2 to 1.59 ± 0.32, the expected survival significantly improved, rising from 12-16 months at the start to 24 months by the end of the five-year monitoring period. It is also noteworthy that the proportion of patients in the high dialysis dose Group 2 grew in the last two years of follow-up. We believe that the benefits of intensified dialysis therapy on patient survival may become apparent after the third year [2]. A potentially insufficient follow-up period might explain why some researchers question the advantages of higher-than-standardized dialysis doses for patient prognosis [2,3,5,21,22].

It is important to recognize that a high dialysis dose can only be deemed effective when patients maintain optimal nutritional status, with nPCR>1.2, according to KDOQI guidelines [1]. In other cases, irrespective of whether spKt/V≥1.5 is achieved, it may be inadequate. This is supported by ROC curve analyses, which indicate that for patients with high dialysis doses and a cutoff spKt/V<1.41, predictors of death include a nPCR <1.2 and serum albumin levels below 35.2 g/L [2]. Research by Ferreira et al. highlighted significant differences in serum albumin, showing that patients with HD≥40 g/L had a markedly lower risk of death (HR 0.23, 95% CI = 0.097-0.541) [9]. A 10-year study by Kato et al. (2013) also reported a higher mortality risk in patients with serum albumin below 38 g/L [2,23]. According to Teixeira et al. (2015), serum albumin is independently linked to low survival rates, while Msaad et al. (2019) discovered that 77.27% of patients who died had low serum albumin levels [2,24,25]. Furthermore, Ebhahimi et al. (2019) reported that every 10 g/L increase in serum albumin corresponded to a 23% increase in survival time for HD patients [26]. Thus, hypoalbuminemia is a significant marker of malnutrition and a strong predictor of mortality in ESRD patients. However, caution is advised when using serum albumin as a nutritional status indicator, as low levels in HD patients may result from increased protein catabolism due to chronic inflammation and other complications of CKD [2,9,23,27]. Therefore, interpreting spKt/V’s predictive value for patient survival requires consideration of nutritional status [2].

Our analysis of underlying causes of death aligns with existing literature, identifying cardiovascular disease (CVD) as the leading cause, followed by catheter-associated sepsis, primarily from staphylococcal infections [2,7,8,28,29]. While cardiovascular mortality has declined in the general population, this trend is not seen in HD patients, where approximately 50% of deaths are attributed to CVD [2,8,28]. This discrepancy can be partially explained by high comorbidity rates and advanced age among HD patients - about 40% are diabetic, with a mean age of around 60 years, and nearly 20% are over 75. Many patients present with significant cardiovascular issues and severe left ventricular hypertrophy at dialysis enrollment [2,7]. Factors like hyperlipidemia, arterial hypertension control, and hyperphosphatemia with secondary hyperparathyroidism should also be considered. The effects and predictive values of these factors have been thoroughly investigated by Liabeuf et al. (2019) in the European study Dialysis Outcomes and Practice Patterns Study (EURODOPPS), which is part of the international cohort study Dialysis Outcomes and Practice Patterns Study (DOPPS) [2,9,30].

Our results suggest that spKt/V≥1.5 serves as a threshold that enhances patient prognosis. Cox regression analysis indicated a twofold increase in mortality risk for patients receiving inadequate dialysis (HR 2.11, 95% CI = 1.32-3.39, p = 0.002), contrasted by a significant reduction in the high dialysis dose Group 2 (HR 0.6, 95% CI = 0.35-1.02, p = 0.051). Notably, the overall mortality rates in our sample rose significantly over the last two years of the study. While this might suggest an increased death risk due to intensified dialysis, it is crucial to note that over 50% of deaths were due to COVID-19 and its complications, a new risk factor that emerged post-2020 (p > 0.05). In a corrected mortality model, the positive impact of high dialysis doses on survival rates is clearly evident [2]. Although our findings contrast with those of the Hemodialysis (HEMO) study, which found no benefits from intensified dialysis or high-flow dialyzers, many recent studies support our conclusions [2,21,22]. Data from Tomo et al. (2021) within the DOPPS study and findings by Marshall et al. (2016) regarding mortality in the HD population of Australia and New Zealand advocate for intensified regimens and the pursuit of high dialysis as a predictor of improved clinical outcomes and survival [2,12,18].

Limitations

This study has several limitations, including the exclusion of pediatric patients, a focus on a five-year period, and the lack of online hemodiafiltration as a method for increasing dialysis doses. Additionally, mortality outcomes in the final two years were impacted by COVID-19 and its complications, and the study only included patients from a single clinic. This requires conducting additional long-term clinical trials to validate the results achieved and develop strategies to improve the quality of life and survival of dialysis patients.

## Conclusions

We believe that the contemporary nephrology community should prioritize reaching a high dialysis dose of spKt/V≥1.5 to improve patient outcomes and overall prognosis. Our results highlight that the advantages of a high dialysis dose are durable over time, but realizing these benefits demands commitment and careful monitoring - not just of adequacy indicators but also of the multiple complications that arise from ESRD.

It is essential to maintain stable nutritional status, targeting an nPCR>1.2 and to diligently monitor serum albumin levels as a key risk factor for mortality, especially in older patients who may face challenges in managing protein catabolism. Implementing this holistic therapeutic approach could lead to improved clinical outcomes, lower healthcare costs, and enhanced quality of life for patients.
